# Supplementary material for: Chronological age estimation from human microbiomes with transformer-based Robust Principal Component Analysis
Source: Commun Biol. 2025 Aug 6;8:1159. doi: 10.1038/s42003-025-08590-y (PMC12328700; doi:10.1038/s42003-025-08590-y)
Supplement: Supplementary file 1 — Supplementary Information [file 42003_2025_8590_MOESM1_ESM.pdf]

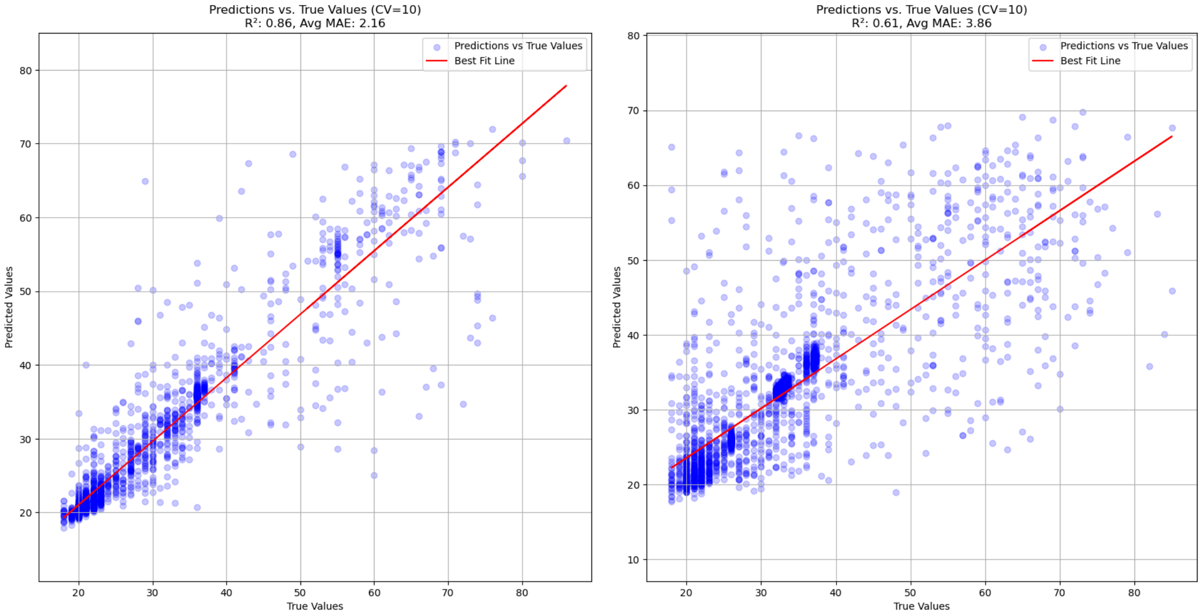

Figure S1: Performance of TRPCA (CV=10) on 16S Skin and 16S Oral samples without grouping samples by host subject for direct comparison with Huang et. al.

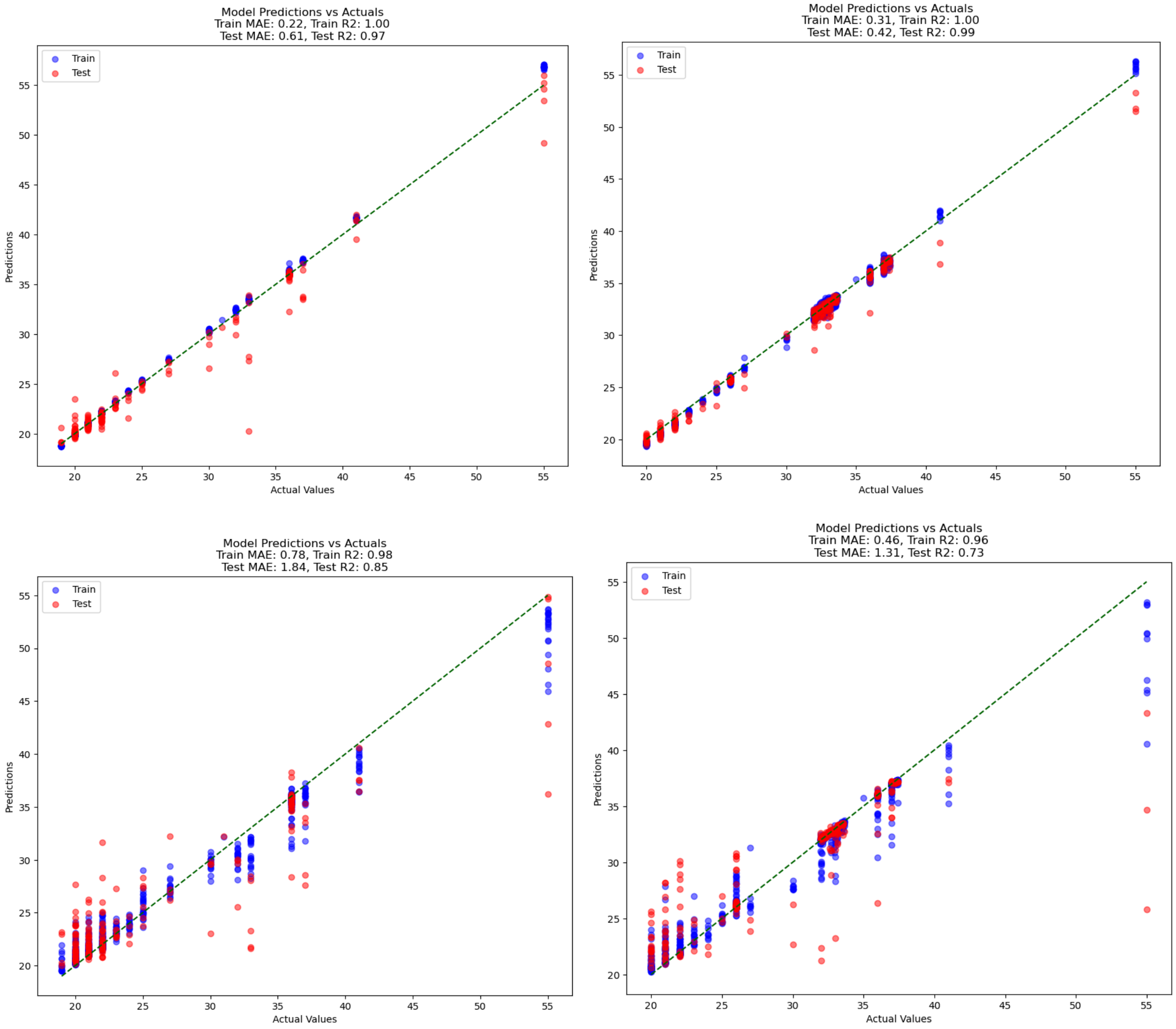

Figure S2: Performance of TRPCA without grouping samples by host subject for the top 40 subjects with greater than 2 samples from the 16S skin and oral datasets (a,b). Samples from each individual were stratified between the 80/20 train and test splits. Deep learning methods such as TRPCA may be well suited for detecting subtle changes in longitudinal data or datasets with multiple samples per subject. Predictions on the same data splits using RF are included for comparison (c, d).

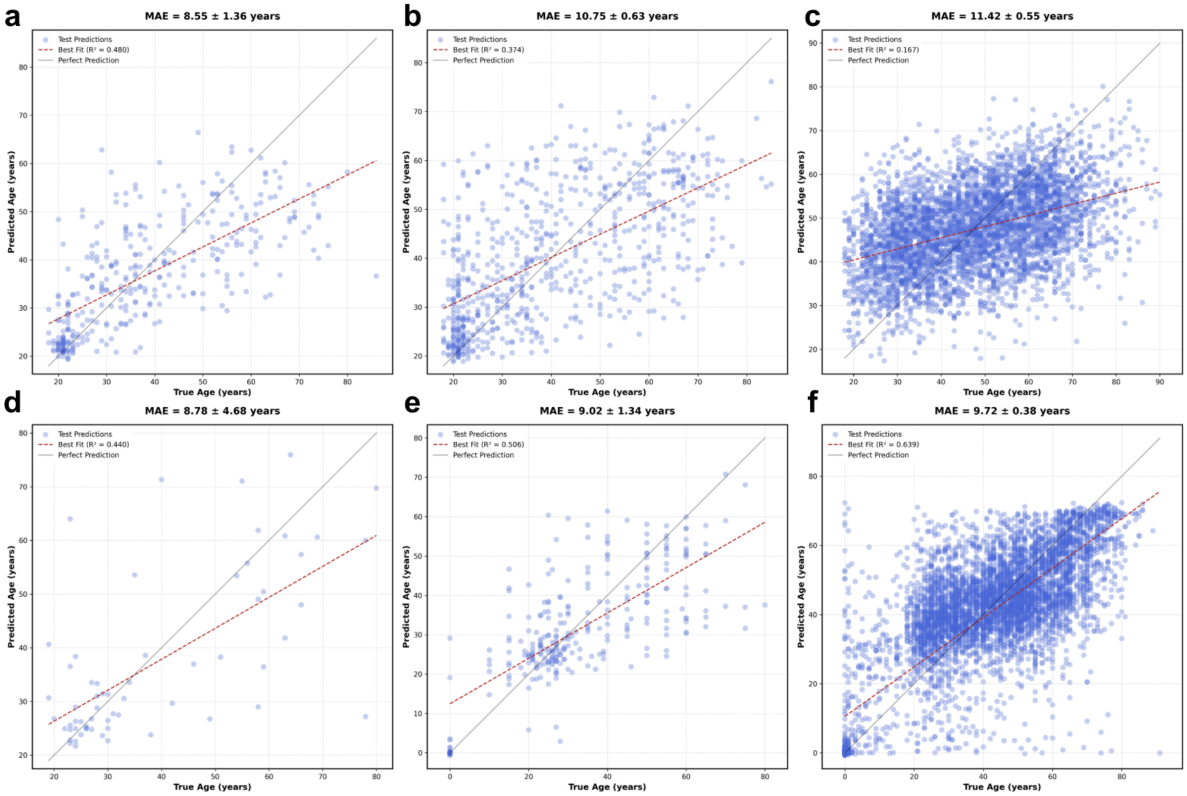

Figure S3: Regression for age prediction by sequencing method (**a-c** for 16S and **d-f** for WGS), and body site (**a,d**; skin, **b,e**; oral, **c,f**; gut), with only one sample per subject. 16S Gut regression remains unchanged as the dataset had only one sample per individual.

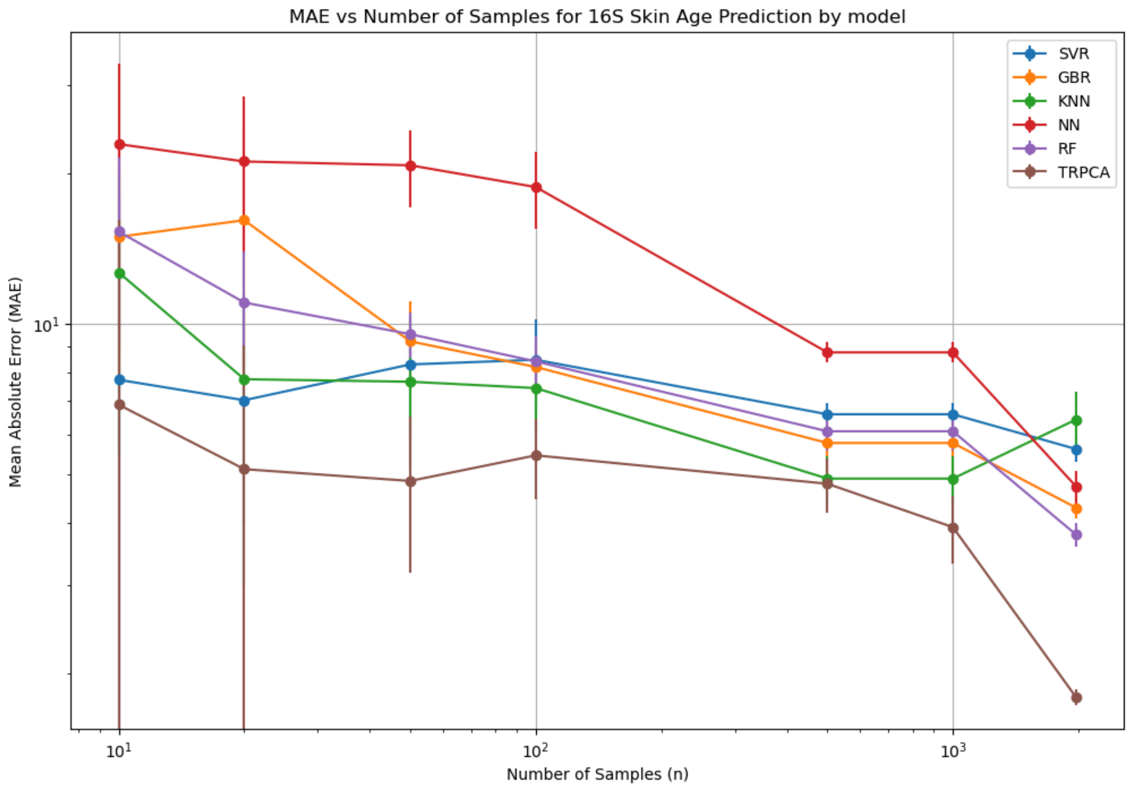

Figure S4: TRPCA (CV=5) outperforms other commonly used ML models for microbiome data analysis. Axes are log scaled for MAE and number of samples.

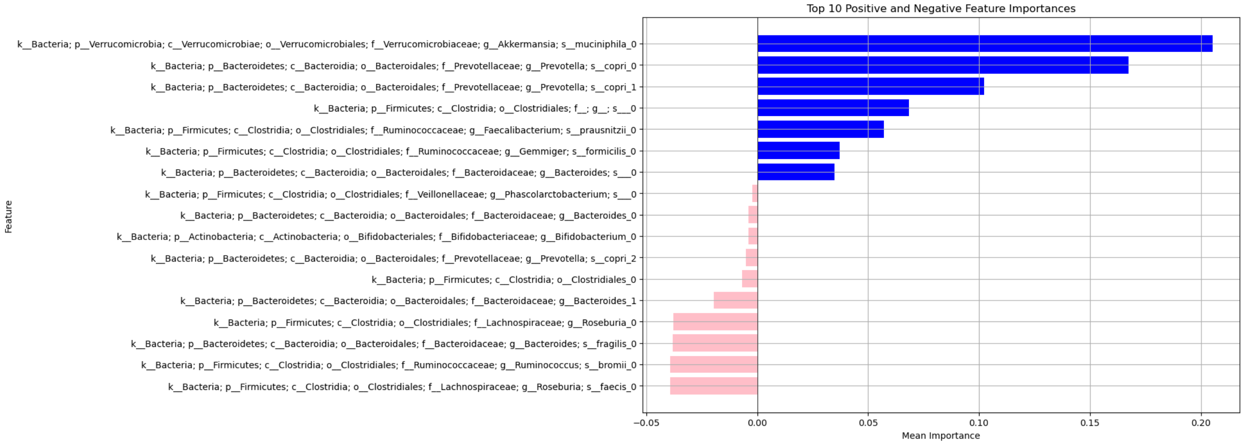

Figure S5: Top 16S Gut microbiome features.

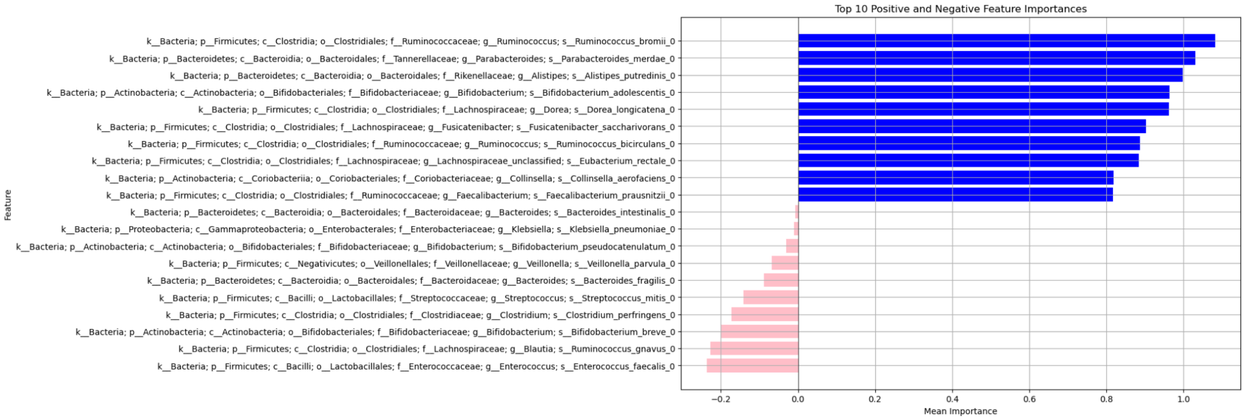

Figure S6: Top WGS Gut microbiome features.

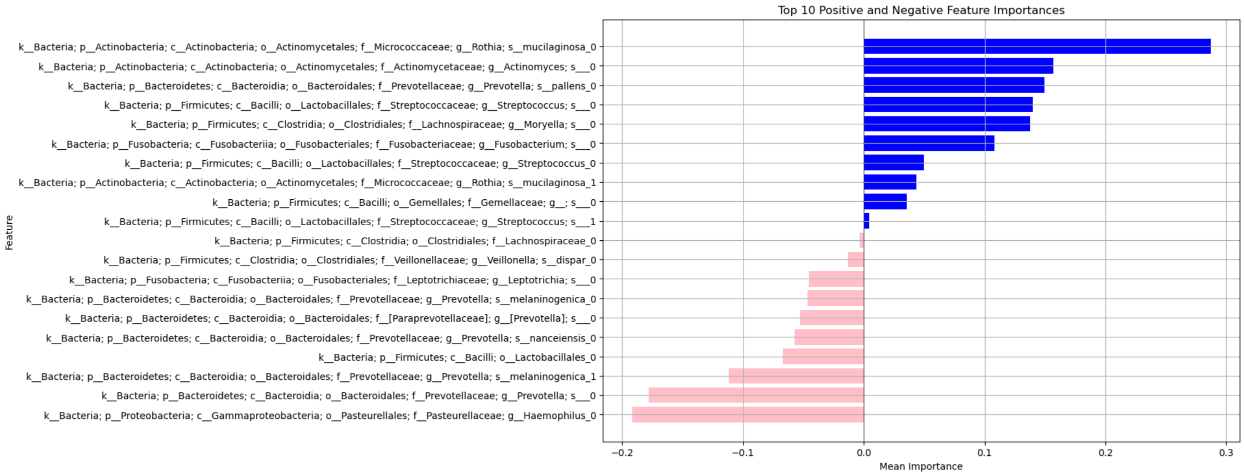

Figure S7: Top 16S Oral microbiome features.

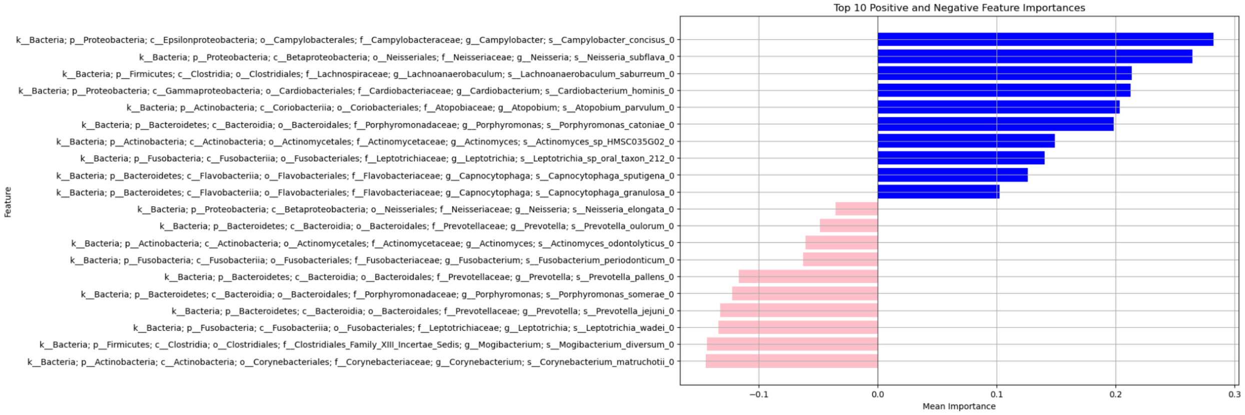

Figure S8: Top WGS Oral microbiome features.

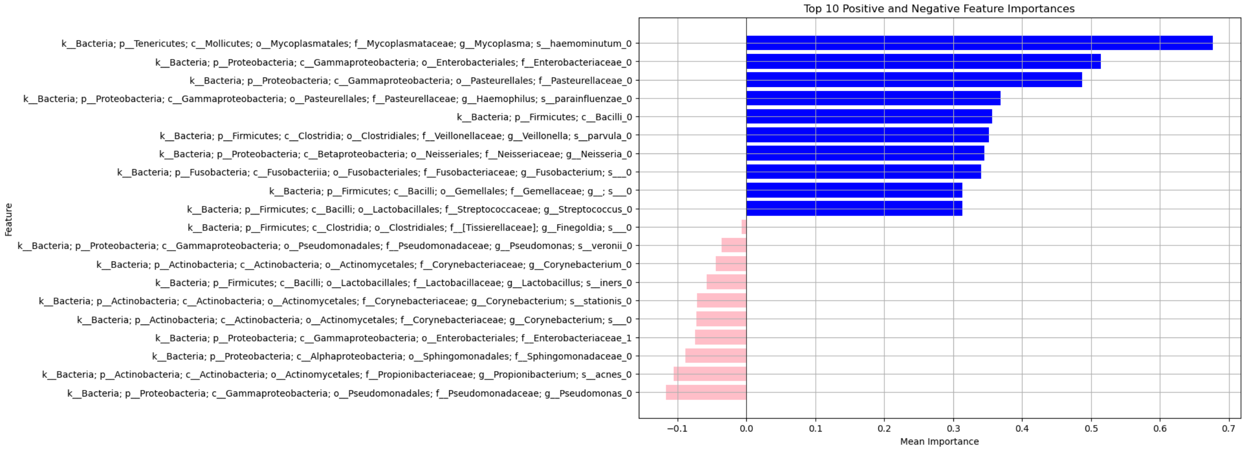

Figure S9: Top 16S Skin microbiome features.

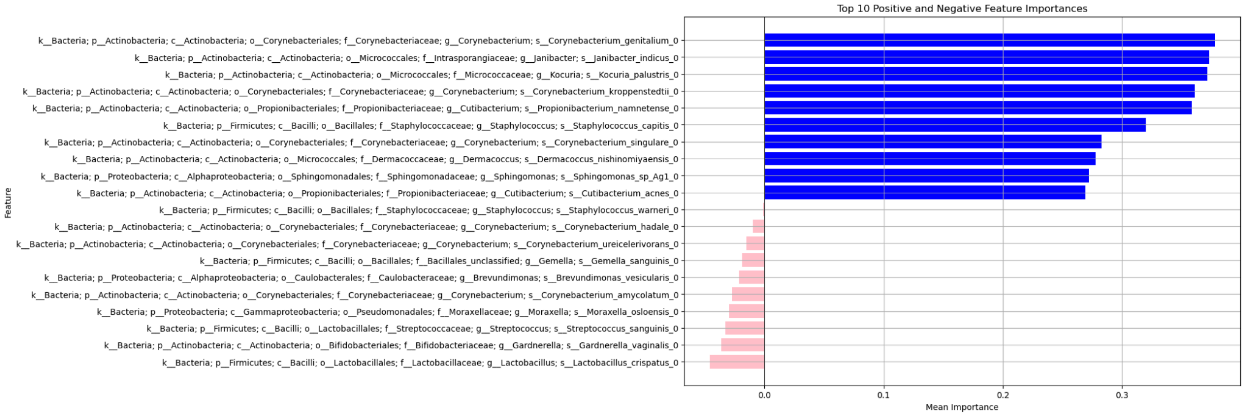

Figure S10: Top WGS Skin microbiome features.

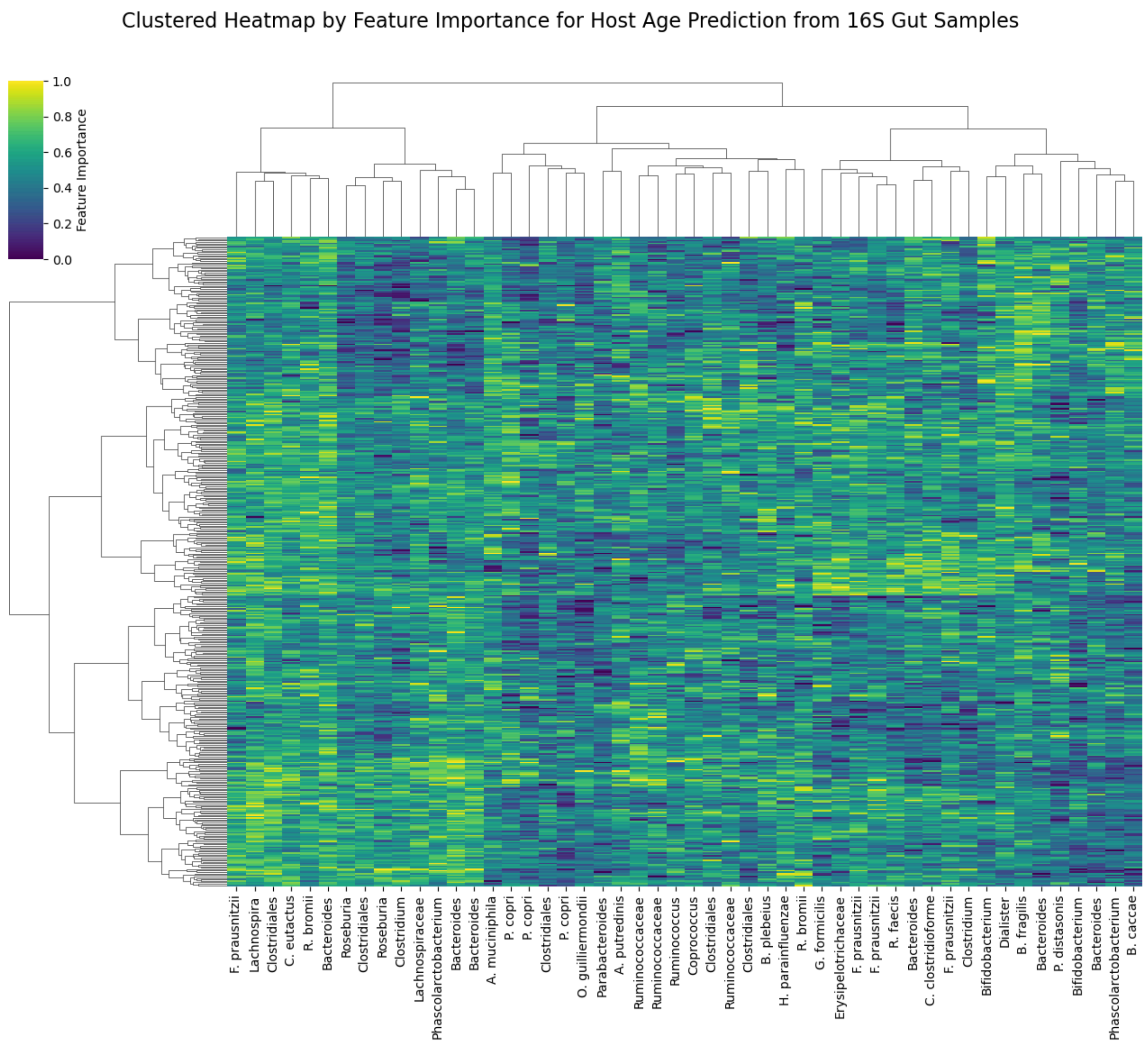

Figure S11: 16S Gut microbiome feature importance heatmap by sample.

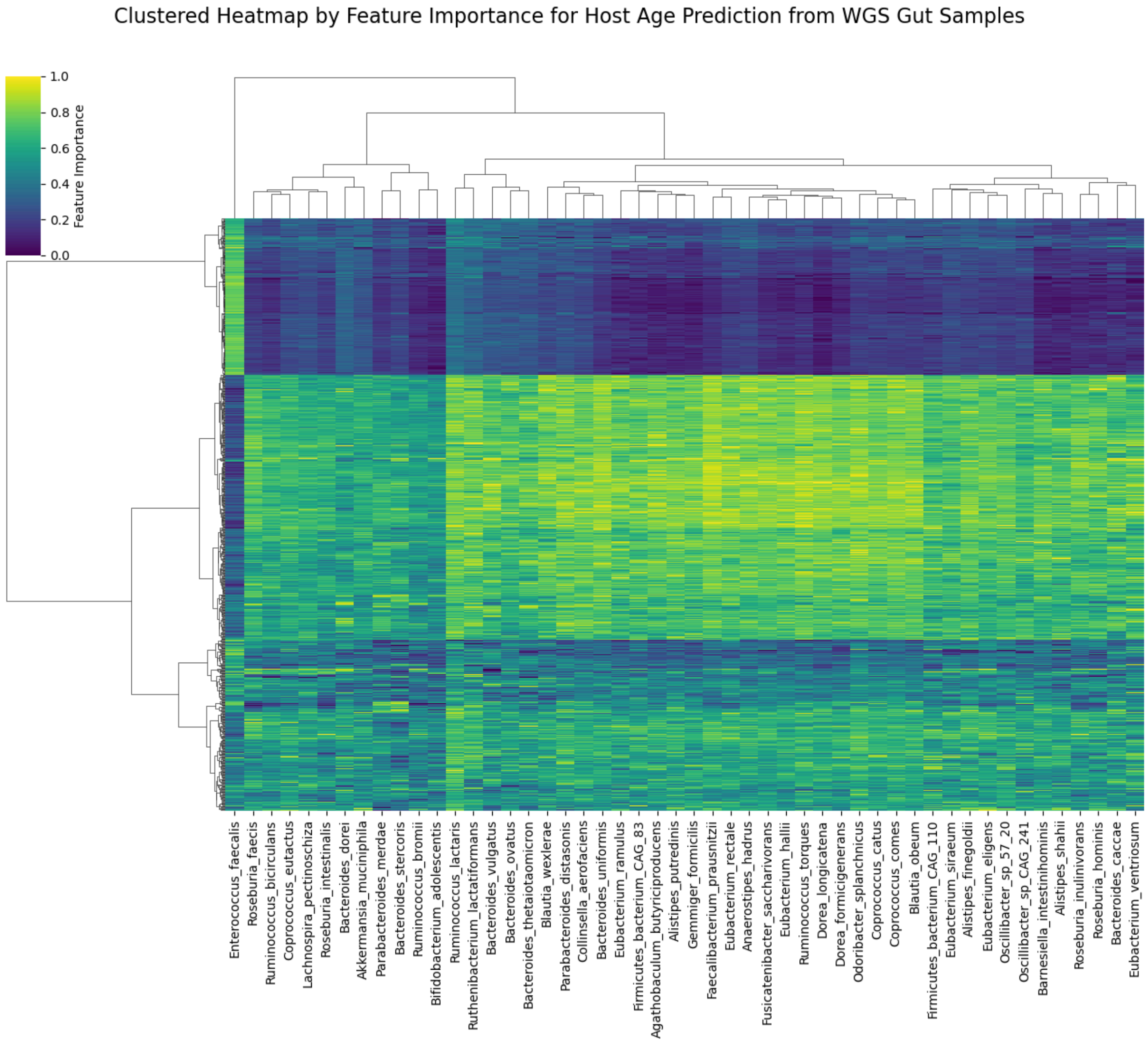

Figure S12: WGS Gut microbiome feature importance heatmap by sample.

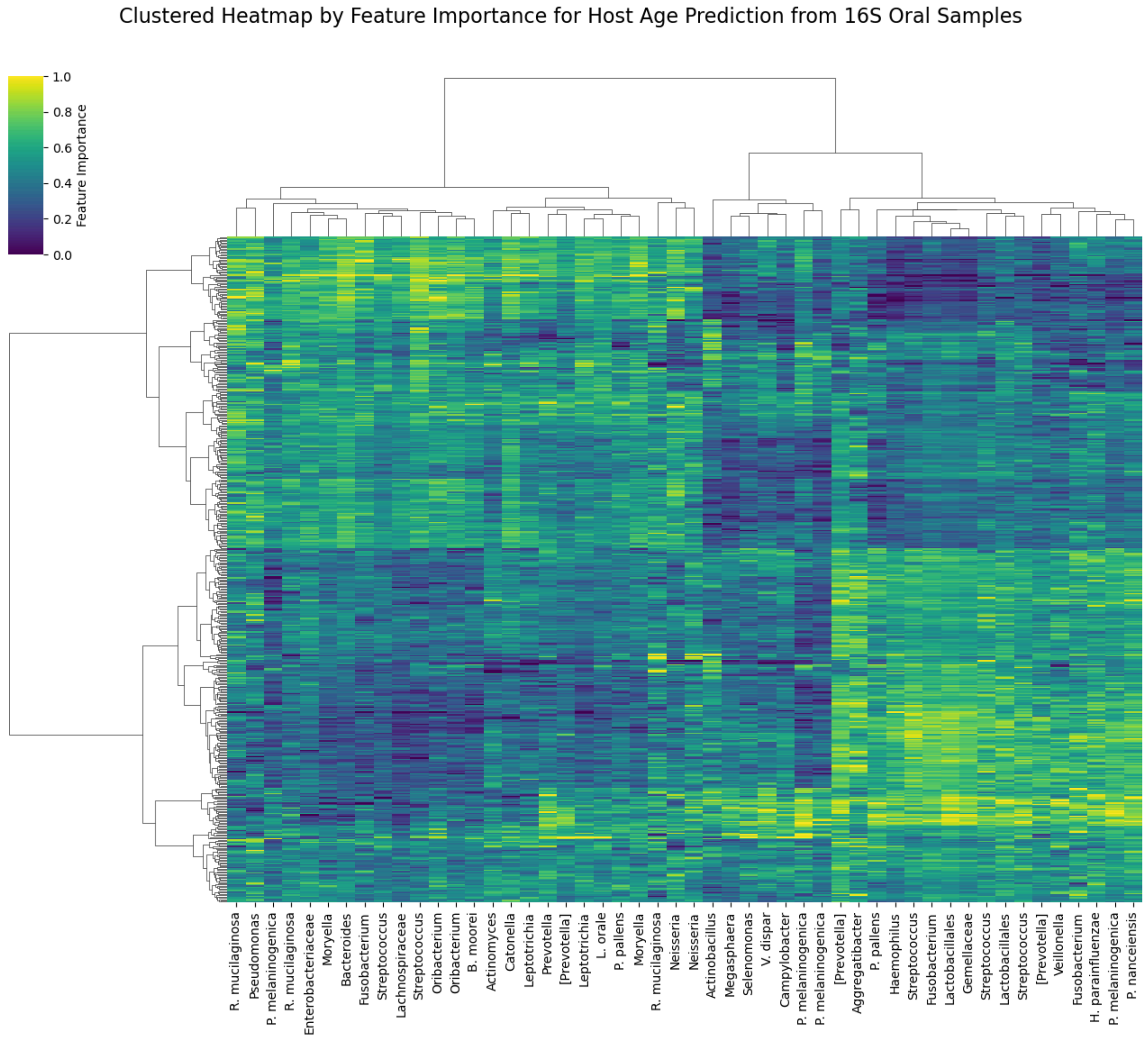

Figure S13: 16S Oral microbiome feature importance heatmap by sample.

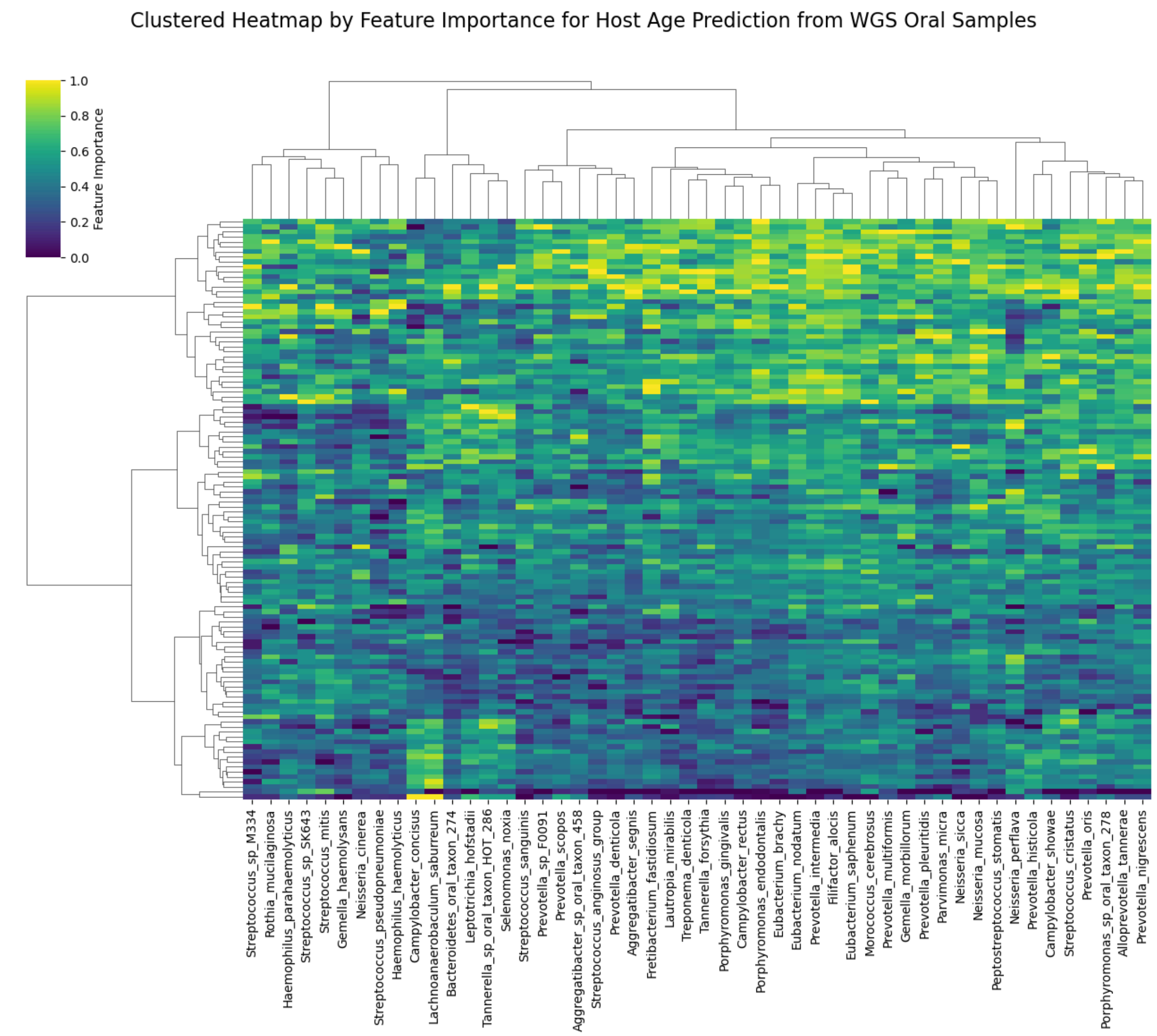

Figure S14: WGS Oral microbiome feature importance heatmap by sample.

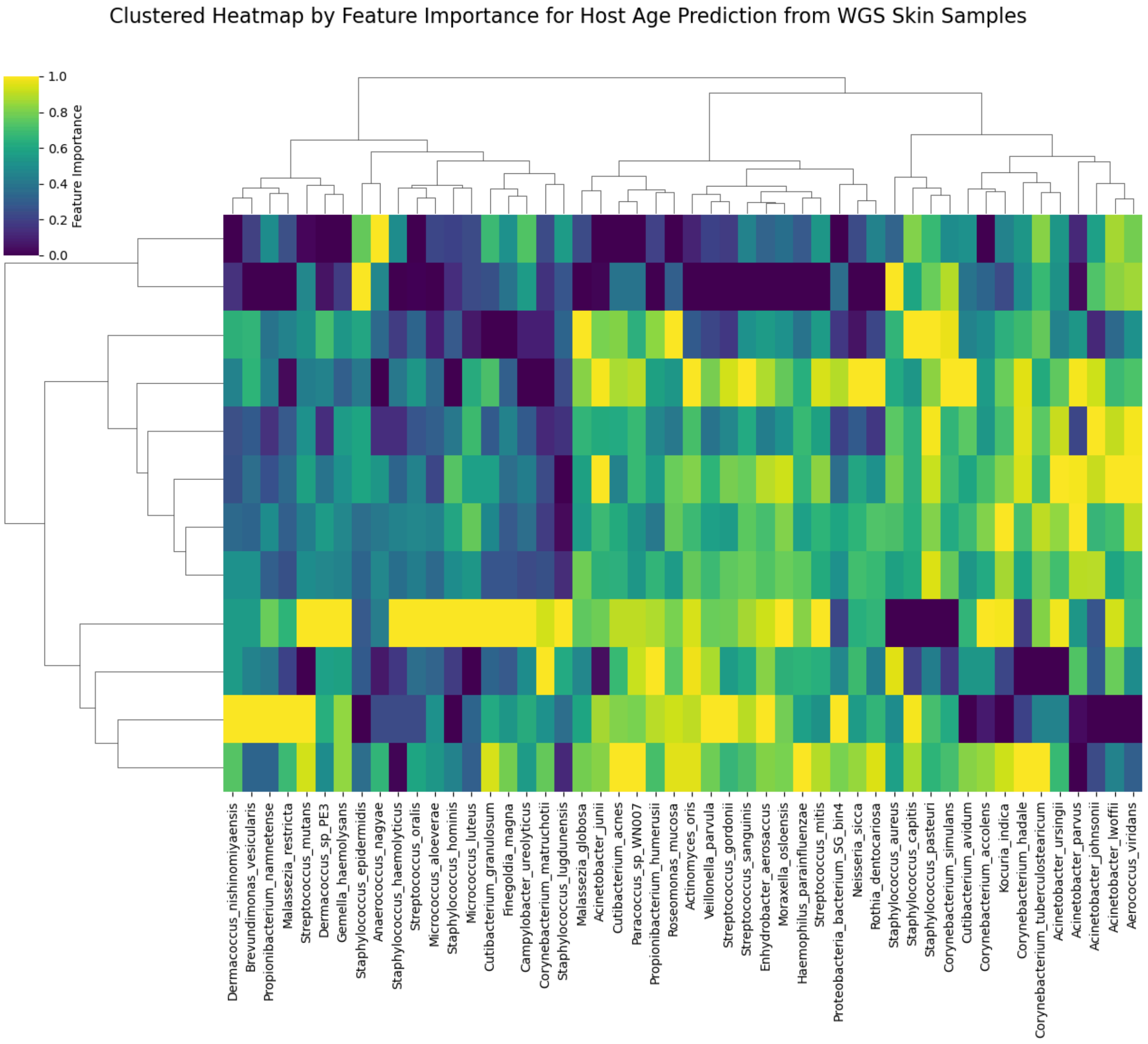

Figure S15: WGS Skin microbiome feature importance heatmap by sample.

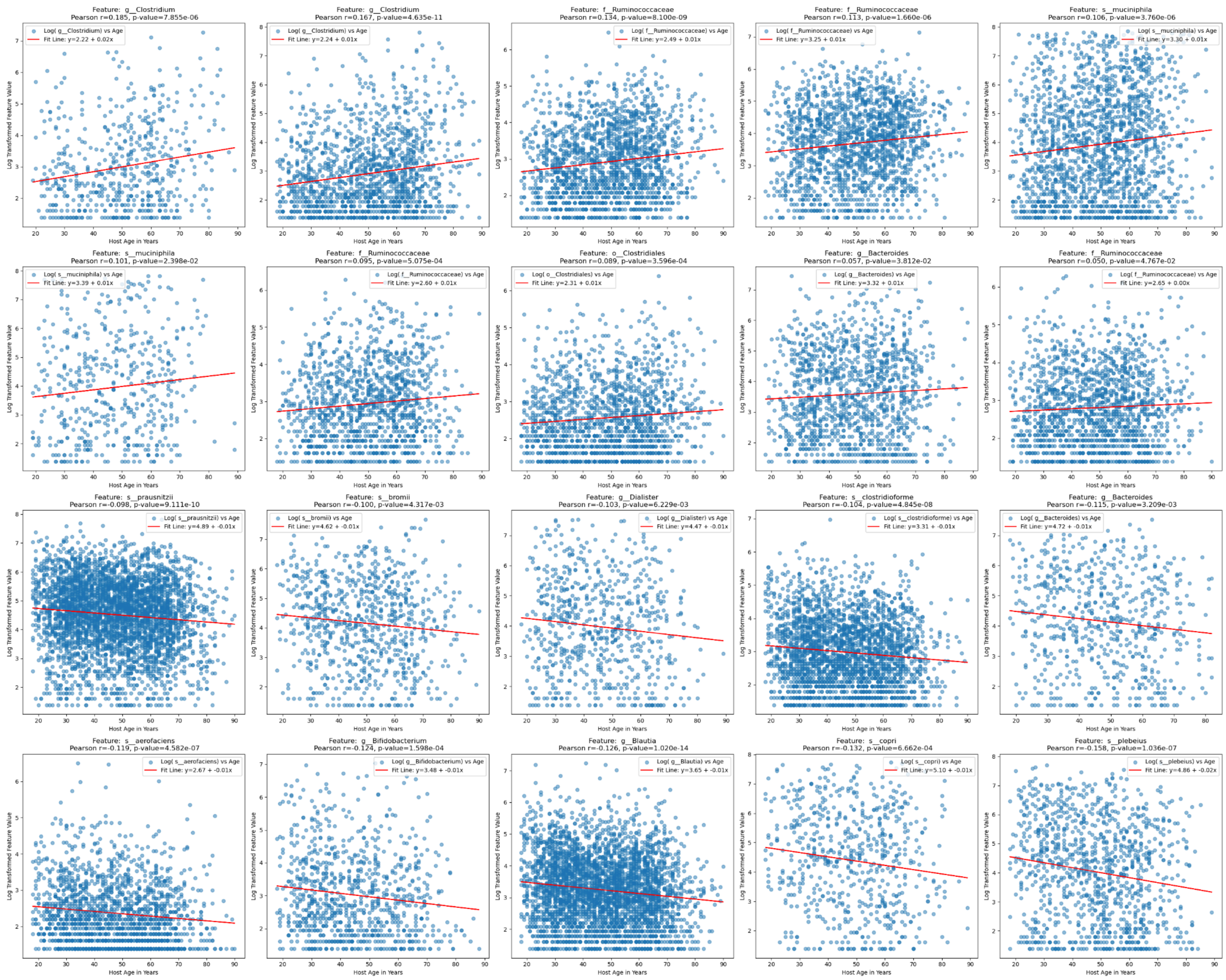

Figure S16: CLR transformed values vs host age for top 10 features correlated with higher host age and 10 features correlated with lower host age for 16S Gut samples.

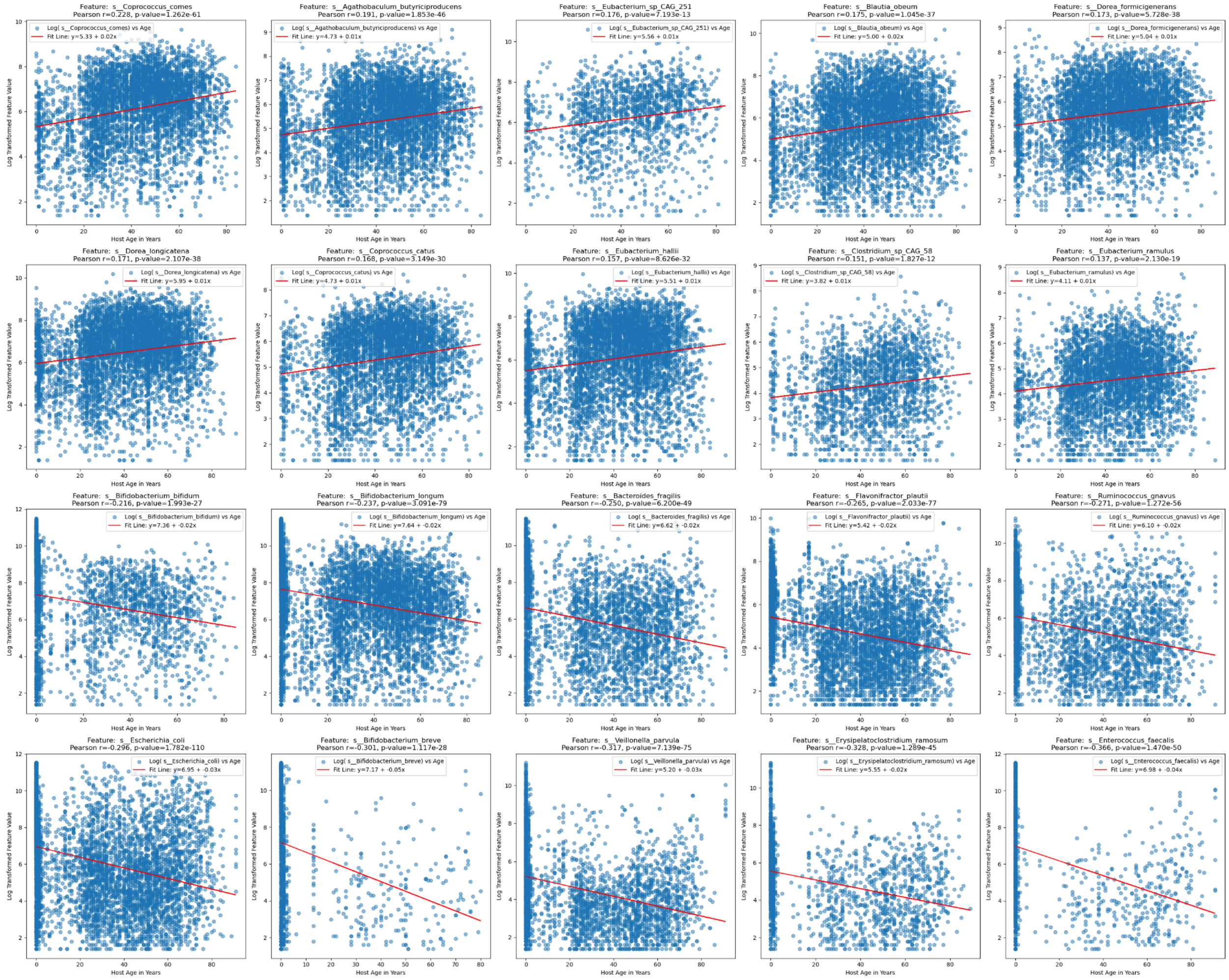

Figure S17: CLR transformed values vs host age for top 10 features correlated with higher host age and 10 features correlated with lower host age for WGS Gut samples.

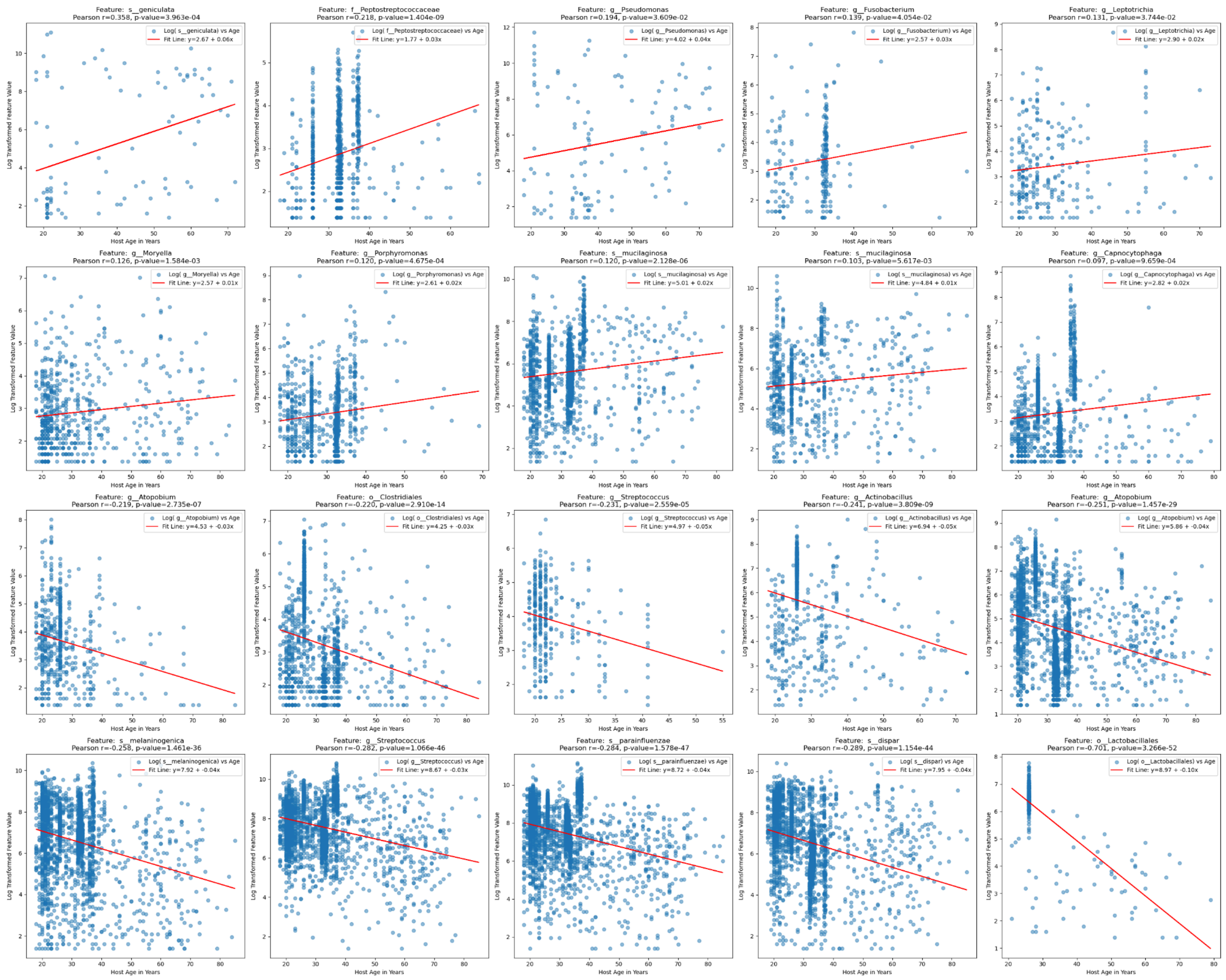

Figure S18: CLR transformed values vs host age for top 10 features correlated with higher host age and 10 features correlated with lower host age for 16S Oral samples.

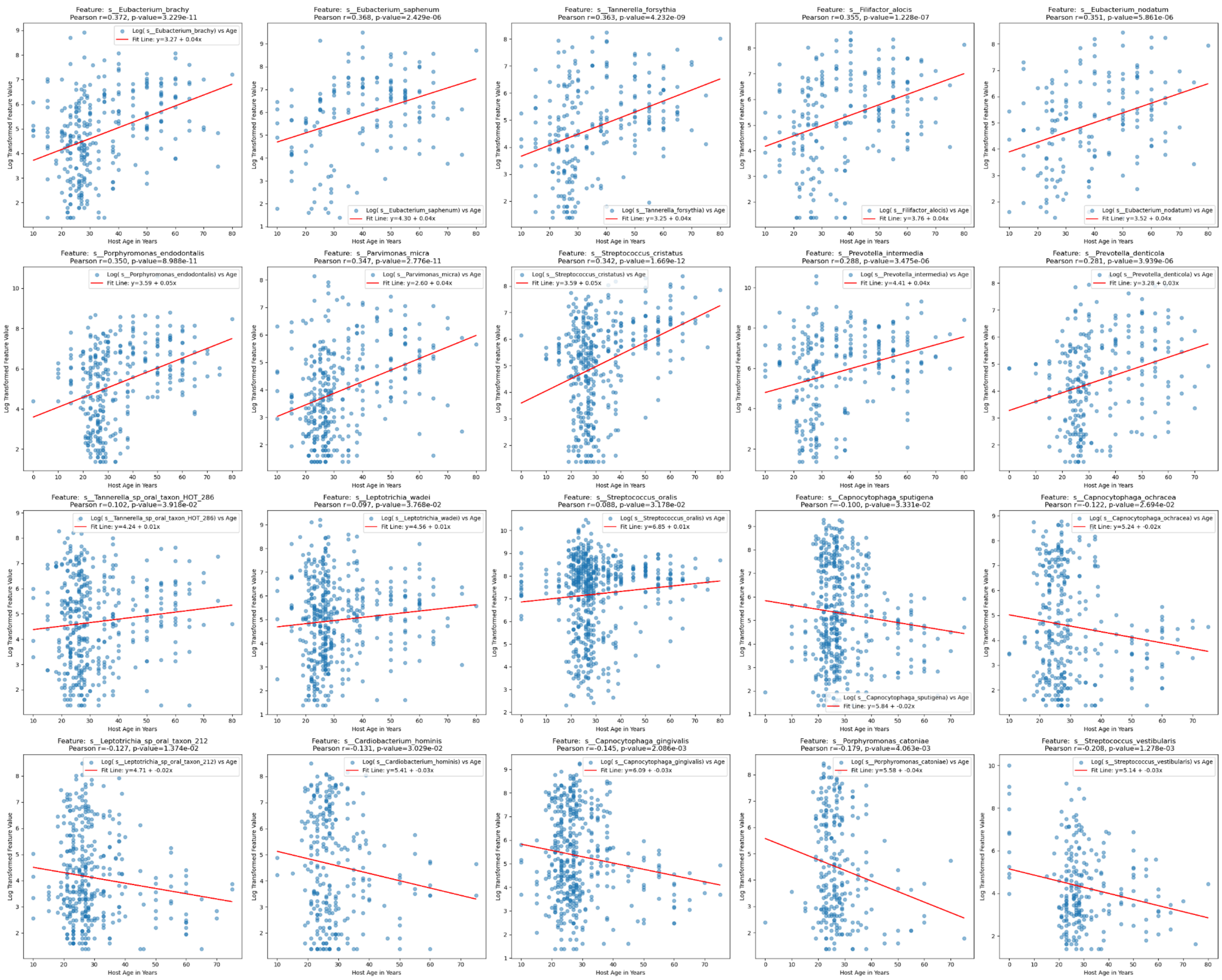

Figure S19: CLR transformed values vs host age for top 10 features correlated with higher host age and 10 features correlated with lower host age for WGS Oral samples.

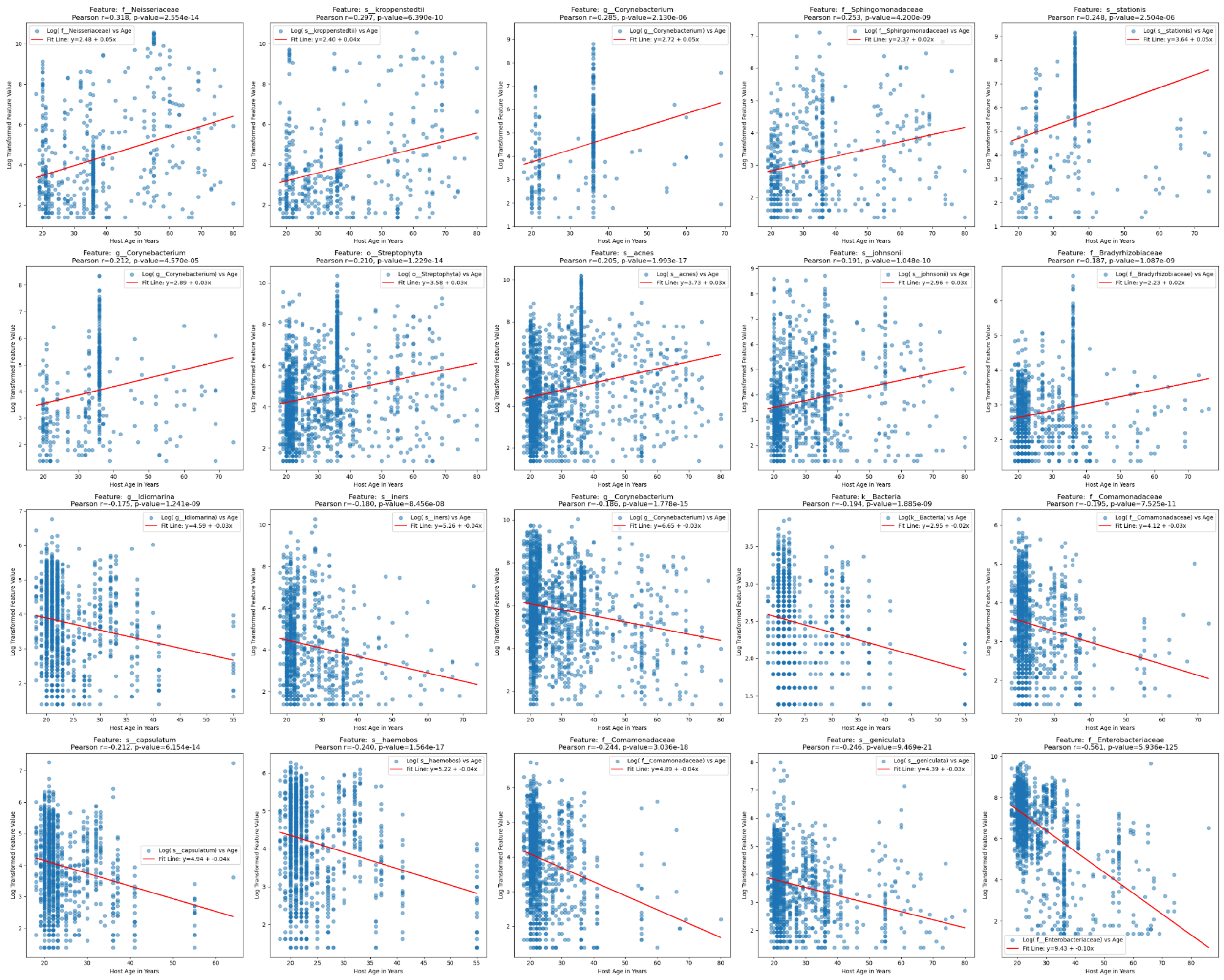

Figure S20: CLR transformed values vs host age for top 10 features correlated with higher host age and 10 features correlated with lower host age for 16S Skin samples.

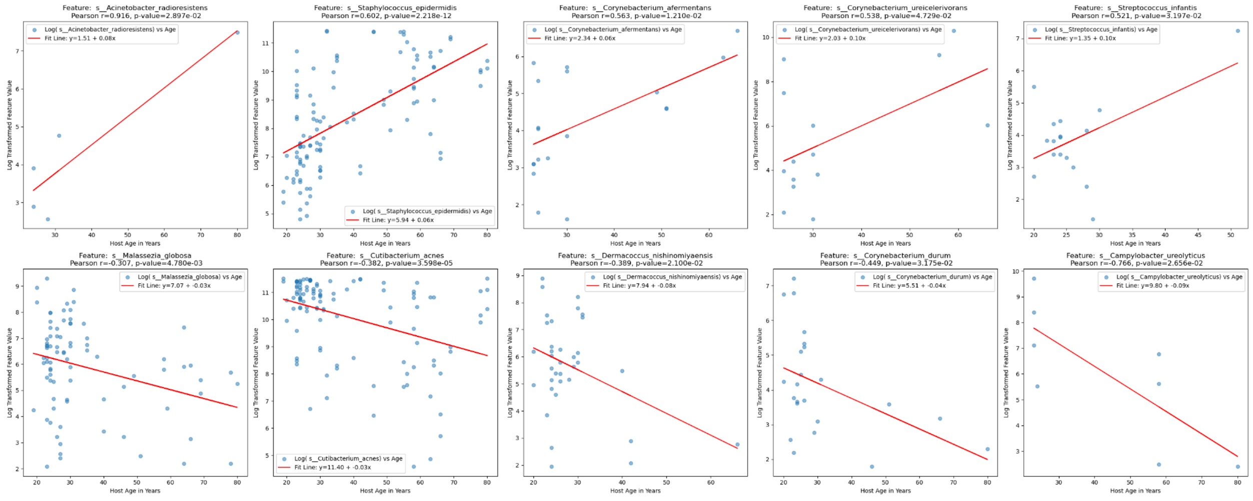

Figure S21: CLR transformed values vs host age for top 5 features correlated with higher host age and 5 features correlated with lower host age for WGS Skin samples.

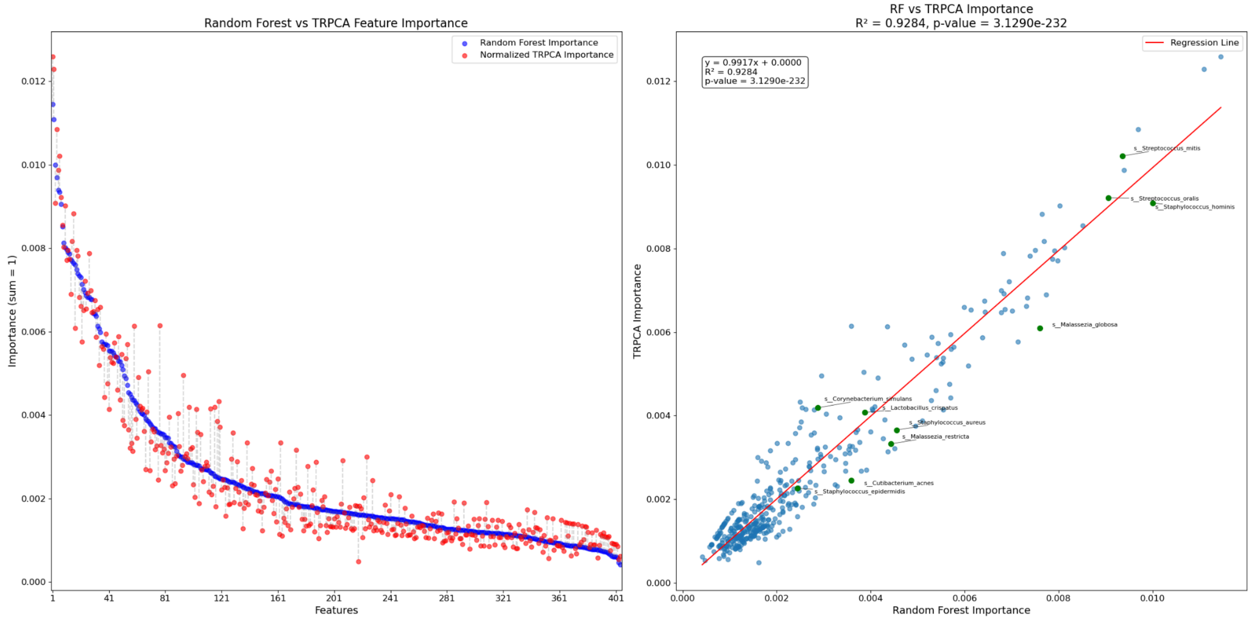

Figure S22: Comparison and regression of feature importances between RF and TRPCA models on WGS Skin microbiome samples. The WGS feature importances are derived from the feature loadings from RPCA for both the RF and TRPCA, as the models are trained on dimensionality reduced data. On the RPCA component level, feature importances show correlation (R2=0.78, p=5.42e-14). Although TRPCA outperforms RF for many regression tasks, there is a strong concordance between important features for host age prediction between model architectures.

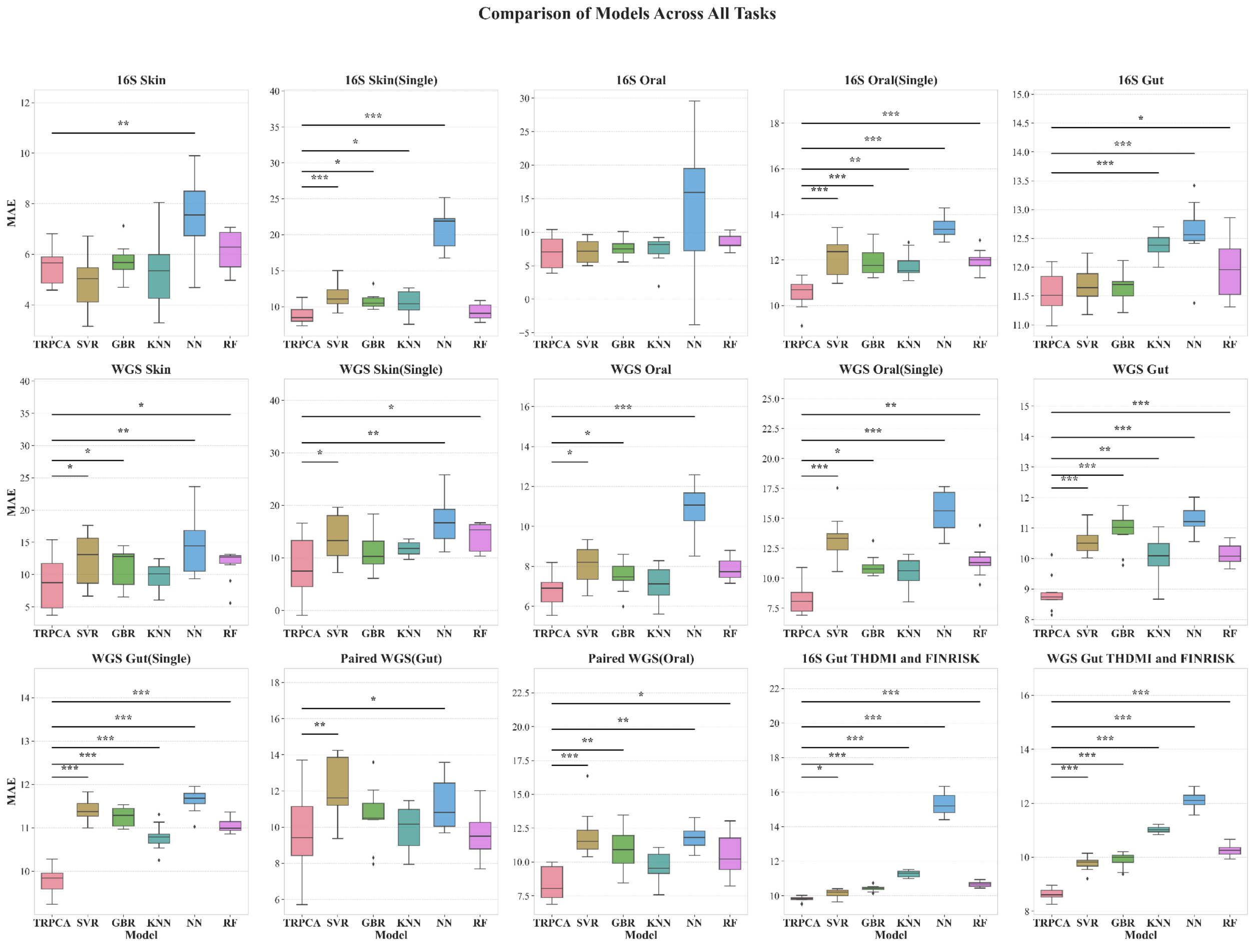

Figure S23: Comparison of model performance between models for each regression task, with t-test for significance between model MAE (CV=10, p-value<0.05 *, p-value<0.01 **, p-value<0.001 ***).

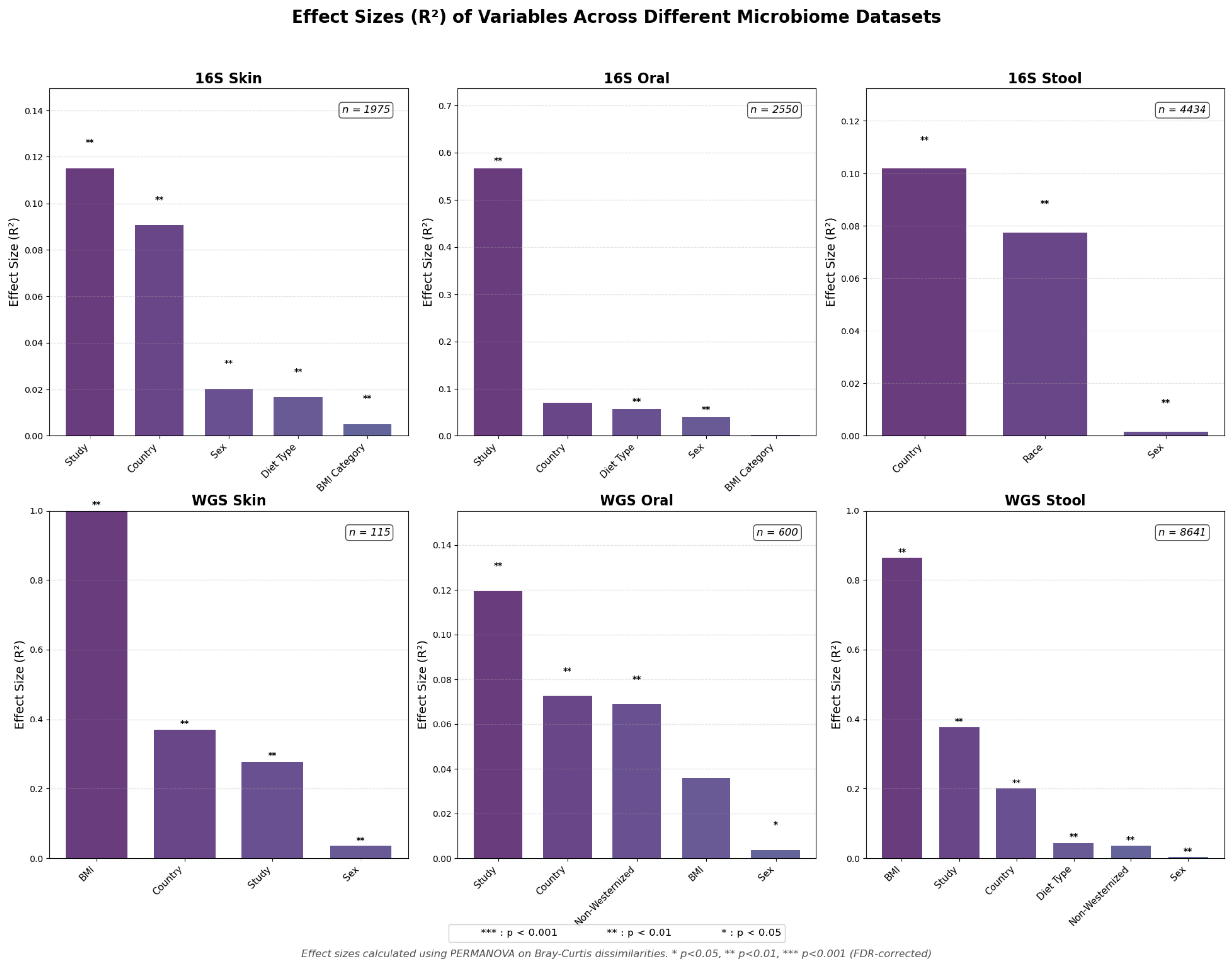

Figure S24: Effect sizes (R^2) of demographic and study variables on microbiome composition across different body sites (skin, oral, stool) and sequencing methods (16S, WGS). Bar heights represent the proportion of microbiome variation explained by each variable, calculated using PERMANOVA on Bray-Curtis dissimilarities, with significance levels indicated by asterisks (* p<0.05, ** p<0.01, *** p<0.001, FDR-corrected).

Data Type	Body Site	Number of Samples	Number of Subjects	Number of Studies	Number of Countries	% Female
16S	Skin	1975	450	4	15	31.1
16S	Oral	2550	724	9	23	41.2
16S	Gut	4434	4434	2	33	56.2
WGS	Skin	115	49	3	7	32.2
WGS	Oral	600	276	5	3	46.5
WGS	Gut	8641	6122	52	27	55

Table S1: Description of datasets used for age regressions.

Table S2: All model performances for age regression tasks.

Table S3: Results of permutation-based sensitivity analysis showing variables that significantly influence age prediction residuals across different microbiome datasets (16S and WGS) from skin, oral cavity, and stool samples, with statistical measures and FDR-corrected p-values.

Table S4: Summary of sensitivity analysis comparing the influence of demographic and study variables on age prediction residuals across six microbiome datasets (16S and WGS sequencing from oral, skin, and stool samples), with values representing statistical significance levels and/or correlation coefficients.
