# Supplementary material for: Chronological age estimation from human microbiomes with transformer-based Robust Principal Component Analysis
Source: Commun Biol. 2025 Aug 6;8:1159. doi: 10.1038/s42003-025-08590-y (PMC12328700; doi:10.1038/s42003-025-08590-y)
Supplement: Supplementary file 3 — Description of Additional Supplementary Files [file 42003_2025_8590_MOESM3_ESM.docx]

Description of Additional Supplementary Files

**File name:** Supplementary Data 1-3

**Description:** Large supplemental tables and the source data behind the figures in the paper.
