# Supplementary material for: Chronological age estimation from human microbiomes with transformer-based Robust Principal Component Analysis
Source: Commun Biol. 2025 Aug 6;8:1159. doi: 10.1038/s42003-025-08590-y (PMC12328700; doi:10.1038/s42003-025-08590-y)
Supplement: Supplementary file 4 — reporting summary [file 42003_2025_8590_MOESM4_ESM.pdf]

nature portfolio

Corresponding author(s): Andrew Bartko

Last updated by author(s): May 16, 2025

Reporting Summary

Nature Portfolio wishes to improve the reproducibility of the work that we publish. This form provides structure for consistency and transparency in reporting. For further information on Nature Portfolio policies, see our Editorial Policies and the Editorial Policy Checklist.

Statistics

For all statistical analyses, confirm that the following items are present in the figure legend, table legend, main text, or Methods section.

n/a	Confirmed	
☐	☒	The exact sample size (*n*) for each experimental group/condition, given as a discrete number and unit of measurement
☐	☒	A statement on whether measurements were taken from distinct samples or whether the same sample was measured repeatedly
☐	☒	The statistical test(s) used AND whether they are one- or two-sided<br>*Only common tests should be described solely by name; describe more complex techniques in the Methods section.*
☐	☒	A description of all covariates tested
☐	☒	A description of any assumptions or corrections, such as tests of normality and adjustment for multiple comparisons
☐	☒	A full description of the statistical parameters including central tendency (e.g. means) or other basic estimates (e.g. regression coefficient) AND variation (e.g. standard deviation) or associated estimates of uncertainty (e.g. confidence intervals)
☐	☒	For null hypothesis testing, the test statistic (e.g. *F*, *t*, *r*) with confidence intervals, effect sizes, degrees of freedom and *P* value noted<br>*Give P values as exact values whenever suitable.*
☒	☐	For Bayesian analysis, information on the choice of priors and Markov chain Monte Carlo settings
☒	☐	For hierarchical and complex designs, identification of the appropriate level for tests and full reporting of outcomes
☐	☒	Estimates of effect sizes (e.g. Cohen's *d*, Pearson's *r*), indicating how they were calculated

Our web collection on statistics for biologists contains articles on many of the points above.

Software and code

Policy information about availability of computer code

Data collection	No custom code was used for the retrieval of data.
Data analysis	Analysis notebooks and TRPCA model repository can be found at https://github.com/tydymy/TRPCA. Additional analyses include use of the following software packages: scikit-learn version: 1.3.0, SHAP version: 0.45.0 , PyTorch version:2.2.0, Gemelli version:0.0.10.

For manuscripts utilizing custom algorithms or software that are central to the research but not yet described in published literature, software must be made available to editors and reviewers. We strongly encourage code deposition in a community repository (e.g. GitHub). See the Nature Portfolio guidelines for submitting code & software for further information.

Data

Policy information about availability of data

All manuscripts must include a data availability statement. This statement should provide the following information, where applicable:

- Accession codes, unique identifiers, or web links for publicly available datasets
- A description of any restrictions on data availability
- For clinical datasets or third party data, please ensure that the statement adheres to our policy

Datasets for 16S skin, oral and gut samples can be found in the Github repository https://github.com/shihuang047/age-prediction . WGS datasets can be retrieved using the code provided at https://waldronlab.io/curatedMetagenomicDataAnalyses/articles/MLdatasets.html and using only the healthy/control subject dataset.

Paired data from THDMI are part of Qiita study 10317 and European Bioinformatics Institute accession number PRJEB11419. Paired samples from the FINRISK dataset are protected with access details available in the European Genome-Phenome Archive under accession number EGAD00001007035.

Research involving human participants, their data, or biological material

Policy information about studies with human participants or human data. See also policy information about sex, gender (identity/presentation), and sexual orientation and race, ethnicity and racism.

Reporting on sex and gender	Gender was considered and reported in the the supplemental table Table S1.
Reporting on race, ethnicity, or other socially relevant groupings	Subject, race, and country of birth were reported and included in Table S1 where applicable and available.
Population characteristics	Subject inclusion criteria were implemented to recruit healthy individuals. Host age was outlined and thoroughly investigated.
Recruitment	Subjects were recruited through protocols outlined in the original studies, which can be identified from the metadata associated with the publicly available data. 16S data and studies in the WGS data may be subject to bias and error from self reporting in surveys.
Ethics oversight	Ethics oversight information for the recruitment and processing of the biological data can located where available though the original publications associated with the publicly available data. See metadata for PMID's and study identification for a given sample.

Note that full information on the approval of the study protocol must also be provided in the manuscript.

Field-specific reporting

Please select the one below that is the best fit for your research. If you are not sure, read the appropriate sections before making your selection.

☒ Life sciences ☐ Behavioural & social sciences ☐ Ecological, evolutionary & environmental sciences

For a reference copy of the document with all sections, see nature.com/documents/nr-reporting-summary-flat.pdf

Life sciences study design

All studies must disclose on these points even when the disclosure is negative.

Sample size	Sample size was determined from the resulting number of samples after filtering based on study criteria.
Data exclusions	WGS samples from the curatedMetagenomicData repository were excluded if they were not labeled as 'healthy' within the associated metadata. 16S samples were originally selected with the exclusion of individuals with IBD, diabetes, or any self reported chronic illness. Pregnant, disabled, and individuals with recent antibiotic treatment were also excluded from 16S data.
Replication	Replication of biological methods are not applicable here; however, cross validation and stratification were implemented in order to assure and demonstrate the average performance of the models, with standard deviations.
Randomization	Cross validation and stratification were used where applicable to assure equal representation of study id, age group, and country of birth where applicable to reduce bias or lack of representation for any given cross validation fold, with random shuffling for training data. Additionally, grouping of samples was used where applicable to avoid longitudinal or multiple samples from any individual being split between the train/validation/test data splits. This was essential as repeated samples from an individual are known to cluster more closely in beta diversity space, when compared to samples from another individual. This could potential lead to bias and poor model generalization due to subject based over-fitting.
Blinding	For this computational analysis, blinding was not necessary.

Reporting for specific materials, systems and methods

We require information from authors about some types of materials, experimental systems and methods used in many studies. Here, indicate whether each material, system or method listed is relevant to your study. If you are not sure if a list item applies to your research, read the appropriate section before selecting a response.

Materials & experimental systems

n/a	Involved in the study
☒	☐ Antibodies
☒	☐ Eukaryotic cell lines
☒	☐ Palaeontology and archaeology
☒	☐ Animals and other organisms
☒	☐ Clinical data
☒	☐ Dual use research of concern
☒	☐ Plants

Methods

n/a	Involved in the study
☒	☐ ChIP-seq
☒	☐ Flow cytometry
☒	☐ MRI-based neuroimaging

Plants

Seed stocks	N/a
Novel plant genotypes	N/a
Authentication	N/a
